# Three-dimensional surface and ultrasound imaging for daily IGRT of prostate cancer

**DOI:** 10.1186/s13014-016-0734-3

**Published:** 2016-12-13

**Authors:** Marco Krengli, Gianfranco Loi, Carla Pisani, Debora Beldì, Giuseppina Apicella, Valentina Amisano, Marco Brambilla

**Affiliations:** 1 Department of Radiotherapy, University Hospital Maggiore della Carità, Via Solaroli, 17-28100 Novara, Italy; 2 Department of Translational Medicine, University of “Piemonte Orientale”, Novara, Italy; 3 Department of Medical Physics, University Hospital Maggiore della Carità, Novara, Italy

**Keywords:** Prostate cancer, Image-guided radiotherapy, 3D-surface imaging, 3D-ultrasound

## Abstract

**Background:**

Image guided radiotherapy (IGRT) is an essential pre-requisite for delivering high precision radiotherapy. We compared daily variation detected by two non-ionizing imaging modalities (surface imaging and trans-abdominal ultrasound, US) to verify prostate patient setup and internal organ variations.

**Methods:**

Forty patients with organ confined prostate cancer and candidates to curative radiotherapy were enrolled in this prospective study. At each treatment session, after laser alignment, all patients received imaging by a 3D-surface and a 3D-US system. The shifts along the three directions (anterior-posterior AP, cranial-caudal CC, and later-lateral LL) were measured in terms of systematic and random errors. Then, we performed statistical analysis on the differences and the possible correlations between the two modalities.

**Results:**

For both IGRT modalities, surface imaging and US, 1318 acquisitions were collected. According with Shapiro Wilk test, the positioning error distributions were not Gaussian for both modalities.

The differences between the systematic errors detected by the two modalities were statistically significant only in LL direction (*p* < 0.05), while the differences between the random errors were not statistically significant in any directions.

The 95% confidence interval of the residual errors obtained by subtracting the random errors detected with surface images to those detected with US was included in the range from −7 mm to 7 mm corresponding to the minimum PTV margin adopted in AP direction in our clinical routine.

**Conclusions:**

From our data, it emerges that setup misalignments measured by surface imaging can be predictive of US displacements after the adjustment for systematic errors. Moreover, surface imaging can detect setup errors predictive of registration errors measured by US. This data suggest that the two IGRT modalities could be considered as complementary to each other and could represent a daily “low-cost” and non-invasive IGRT modality in prostate cancer patients.

## Background

Accurate and reproducible patient setup is a prerequisite to correctly deliver fractionated radiotherapy for prostate cancer. To correct for daily setup errors and inter-fraction organ motion, image guided radiation therapy (IGRT) is used, allowing for decreased safety margins and reduced normal tissues irradiation.

IGRT can be performed by imaging modalities using ionizing radiations such as cone beam computed tomography (CBCT) [1] or electronic portal imaging device (EPID) with fiducials [2, 3], or by imaging techniques not delivering ionizing radiations such as ultrasounds (US) [4, 5], electromagnetic transponders [6] and surface imaging systems [7–9]. Some IGRT modalities detect target position accounting for the overall localization errors without distinguishing external patient setup or internal organ motion (US and electromagnetic transponders), other IGRT modalities can detect only surrogates of setup changes (surface fiducial based imaging systems, bone or other anatomical landmarks in EPID imaging) and others can capture both external setup and internal organs variations (CBCT). The interest to distinguish external setup and internal target variations resides in the possibility to identify and correct each error component.

As a matter of fact, CBCT with fiducials is considered the gold standard to detect both setup and internal organ variations but it is not usually performed daily in long course treatments because of the non-negligible dose of radiation to the patients and staff workload.

Compared to CBCT, surface imaging systems showed mean positioning errors in the range of 0.1 − 4.0 mm whereas 3D-US systems showed mean systematic errors in the range of 1.3–2.5 mm and random errors in the range of 2.3-2.7 mm [10, 11].

In a previous study, we employed the surface imaging system AlignRT (VisionRT, London, UK) to analyze inter-fraction setup variations for prostate cancer [8]. We observed that measurements by this system were highly reproducible and correlated with the setup errors detected by EPID. Similar data were reported by an analogous study comparing AlignRT with digital portal images [9].

In this work, we compared daily setup variations observed by two IGRT modalities relying on surface imaging by AlignRT and trans-abdominal US by Clarity (Resonant Medical, Elekta, SE) in a cohort of patients treated for prostate cancer who underwent also regular quality assurance (QA) procedure by portal imaging. The aim of the study was to verify the consistency of the positioning errors registered by the two modalities and to analyze whether the localization errors measured by AlignRT could be correlated with those measured by Clarity.

## Methods

Forty patients with organ confined prostate cancer, staged cT1c–cT3b N0, median iPSA 10.2 ng/ml and aged 59–81 years (mean 73 years) were enrolled in the study after adequate informed consent and following our institutional rules. Patients were candidates to receive curative radiotherapy to a total dose of 76–78 Gy with daily conventional fractionation of 2 Gy.

All patients underwent simulation by helical CT-scan (Lightspeed, General Electric, Milwaukee, WI, USA) in supine position, using a leg immobilization system (Combifix-Sinmed, Civco, Kalona, IA, USA), with contiguous slices of 3 mm thickness from L4 to 2 cm below the ischeal tuberosities. To optimize reproducibility, patients followed a preparation protocol with empty rectum and full bladder before CT-simulations and before each treatment session. Three skin tattoos, two laterals and one anterior, were marked for position verification by alignment to a laser system. CT data were transferred to the treatment planning system (TPS) Pinnacle (Philips, Eindhoven, The Netherlands) by a local network and target volumes and organs at risk were outlined. Clinical target volume (CTV) was defined as prostate ± seminal vesicles according to clinical and imaging data. Planning target volume (PTV) was obtained by adding 10 mm margin in all directions, except toward the rectum where the margin was set to 7 mm.

During the simulation session, we acquired 3-dimensional (3D) US prostate scan with the Clarity system installed in the CT-simulation room. The Clarity system acquires 3D-US pelvic data with a 2D abdominal US probe outfitted with positional sensors, which is swept across the patient’s region of interest. An infrared camera is used to track these sensors so that the position and orientation of each 2D image may be determined in order to reconstruct a 3D dataset coregistered with the CT dataset [12]. In this regard, the radiotherapy plan, including contours of CTV, PTV and organs at risk (OARs), is imported to Clarity workstation and coregistered with US reference image. The contour of the prostate, based on ultrasound images, is then outlined to create a reference volume to be used for comparison at each treatment session. The CT-US image fusion is performed by a dedicated system implemented in the Clarity workstation.

Treatment was delivered by a linear accelerator Clinac DBX (Varian, Milpitas, CA, USA) equipped with a 120 leaves multileaf collimator and an amorphous silicon electronic portal imaging device (EPID). The treatment room is equipped with both Clarity and AlignRT systems. AlignRT is a commercially available 3D-surface image registration system and the main aspects of its use and performances were described in previous papers [8, 13]. The procedure for surface registration, image acquisition, and comparison with the reference image typically last less than 40 s and the procedure for 3D-US target verification about 120 s.

At each treatment session after alignment by lasers on skin tattoos, the surface image of the abdominal and pelvic region was acquired by the AlignRT system and coregistered with the body reference surface segmented from the simulation CT-scan. Afterwards, a radiation oncologist trained in US prostate imaging, acquired pelvic US image with Clarity system and compared this US image with the reference one by means of prostate coregistration. Bladder filling was specifically checked and whenever insufficient the patient was invited to drink water. For both imaging modalities, AlignRT and Clarity, the shifts on the 3 patient axes anterior-posterior (AP), cranial-caudal (CC) and later-lateral (LL) were recorded.

Before starting irradiation, portal imaging was performed daily for the first five sessions and then weekly to cross-validate setup accuracy with bone anatomy matching. According to our procedure, the patient was repositioned and the setup re-verified in case of misalignment >4 mm. At each following treatment session, the same procedure was performed for all patients and data were recorded in a data base.

In our series, positioning errors detected by AlignRT and by Clarity in the frame of reference of the treatment room have different meanings. By AlignRT, the displacement of the body surface was used as a surrogate of target position and can be considered as a “fiducial registration error”. By Clarity, the target was directly monitored by the IGRT system and the positioning errors are the target registration errors. The “fiducial registration error” that is based on patient surface mainly depends on the setup errors and possible patient shape changes, while the target registration error is affected by setup errors and by organ motion due to bladder and rectal filling while.

By statistical analysis, the normality Shapiro Wilk (SW) test was applied to study the distribution of AlignRT and Clarity measurements. Then, we specifically investigated whether the positioning errors measured by AlignRT could correlate to the target error measured by Clarity. The errors were analyzed by means of descriptive statistics and decomposed in their systematic and random components. The Wilcoxon test was used to verify statistical significance of the differences between the two populations. A *p* value <0.05 was considered as statistically significant.

## Results

**Fig. 1:**
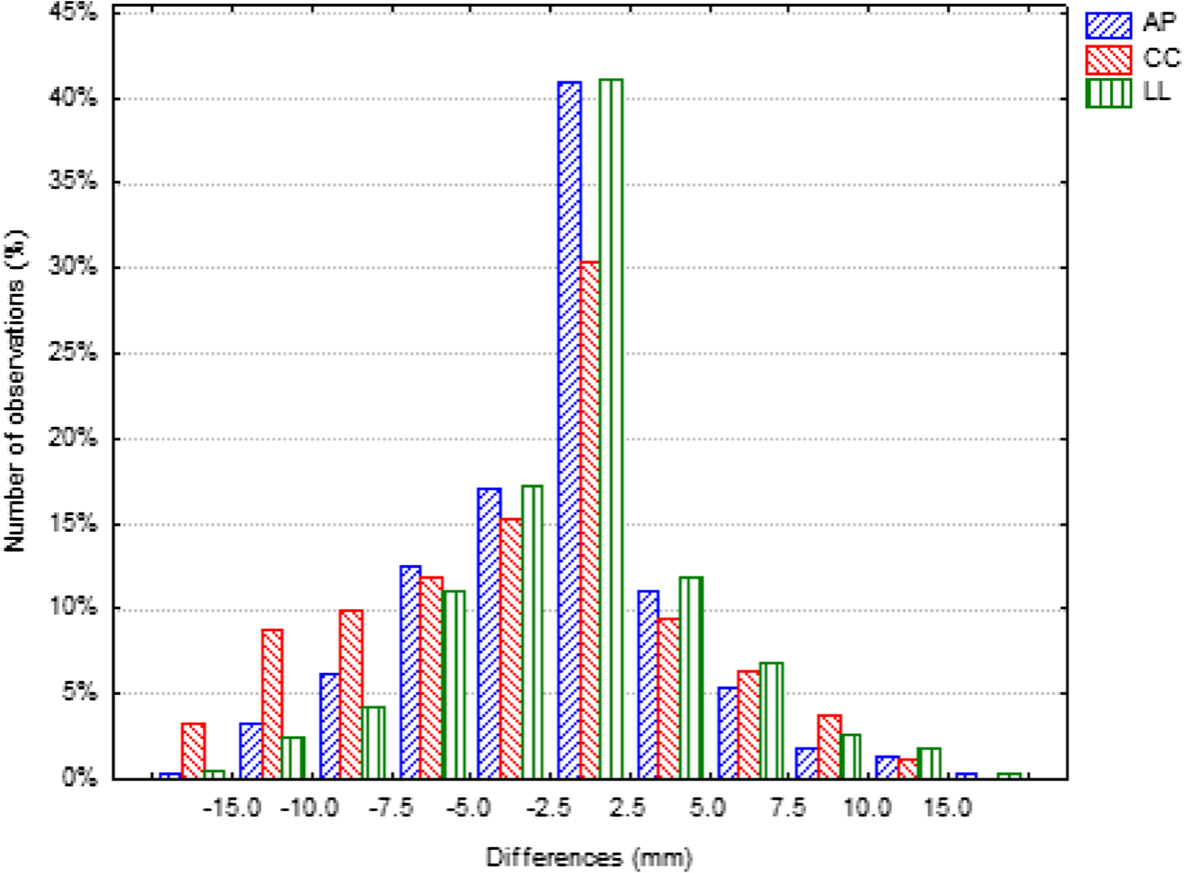
Results as histograms for the frequency distribution of the differences between the paired positioning errors detected by AlignRT and Clarity along the three main axes

For each IGRT modality, AlignRT and Clarity, 1318 acquisitions were collected and analyzed. The mean, the standard deviation (SD), the minimum and maximum values of the positioning errors detected by AlignRT and Clarity along the main axes are reported in Table 1. According to SW test the collected data were not normally distributed and the differences between the positioning errors detected by two imaging systems resulted statistically significant (*p* < 0.001) at the Wilcoxon test for each component along the main axes. The mean, the standard deviation and the minimum and maximum values of the daily positioning error differences between the two modalities are reported in Table 2 and the histograms of the frequency of these differences are reported in Fig. 1.

**Table 1:** Descriptive statistic (mean, standard deviation, maximum and minimum values) of positioning errors (mm) along the three main axes by the two IGRT modalities, AlignRT and Clarity

	AP		CC		LL	
	AlignRT	Clarity	AlignRT	Clarity	AlignRT	Clarity
Mean	1.8	0.6	3.1	0.6	0.7	-0.0
SD	3.3	5.0	4.4	5.1	2.6	4.9
Max	12.0	20.5	21.2	18.2	10.5	24.3
Min	-9.1	-23.8	-18.1	-49.1	-9.4	-17.6

**Table 2:** Descriptive statistic (mean, standard deviation, maximum and minimum values) of the daily displacement differences (mm)

	AP	CC	LL
Mean	-1.2	-2.6	-0.7
SD	4.9	6.4	5.0
Max	18.0	15.9	22.1
Min	-25.8	-48.8	-22.5

In terms of regular QA checks at the setup procedure, portals showed shifts always inferior to 4 mm along the three main axes compared to DRRs after alignment by lasers and surface imaging.

The analysis of the systematic errors for each imaging modality showed that the mean systematic error detected by AlignRT was 2.2 mm (SD = 3.4 mm) in AP direction, 3.1 mm (SD = 3.7 mm) in the CC direction and 0.7 mm (SD = 1.9 mm) in the LL direction and the mean systematic error detected by Clarity was 3.0 mm (SD = 3.1 mm) in AP direction, 2.2 mm (SD = 1.3 mm) in CC direction and −0.1 mm (SD = 1.0 mm) in LL direction.

**Fig. 2:**
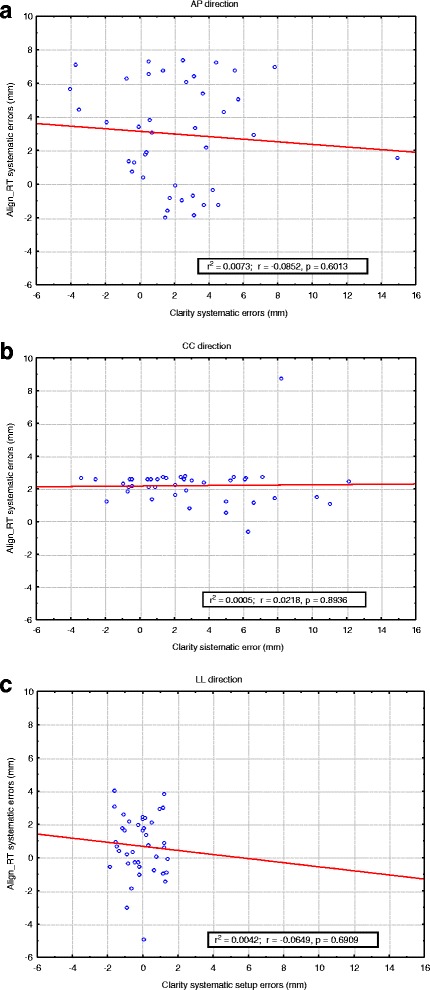
The graphs (**a**, **b**, **c**) show that the systematic errors detected by AlignRT and Clarity are not correlated. The *p* values for the linear regression resulted 0.6013, 0.8936, and 0.6909 respectively for AP, CC, and LL direction

The Wilcoxon test showed that differences between the systematic errors detected by the two modalities were not statistically significant in AP (*p* = 0.288) and CC (*p* = 0.397) directions, but they were in the LL direction (*p* = 0.019). No statistically significant correlation was found between the systematic errors calculated by the two modalities as shown in Fig. 2a, b, c.

The mean random error detected by AlignRT was −0.4 mm (SD = 3.0 mm) in AP direction, 0.1 mm (SD = 2.50 mm) in the CC direction and 0 mm (SD = 1.9 mm) in the LL direction. The mean random error detected by Clarity was 0.1 mm (SD = 3.5 mm) in AP direction, 0.3 mm (SD = 3.7 mm) in CC direction and −0.1 mm (SD = 3.2 mm) in LL direction. The Wilcoxon test showed that the differences between the random errors detected by the two modalities were not statistically significant in AP (*p* = 0.056), CC (*p* = 0.177) and LL directions (*p* = 0.371).

**Fig. 3:**
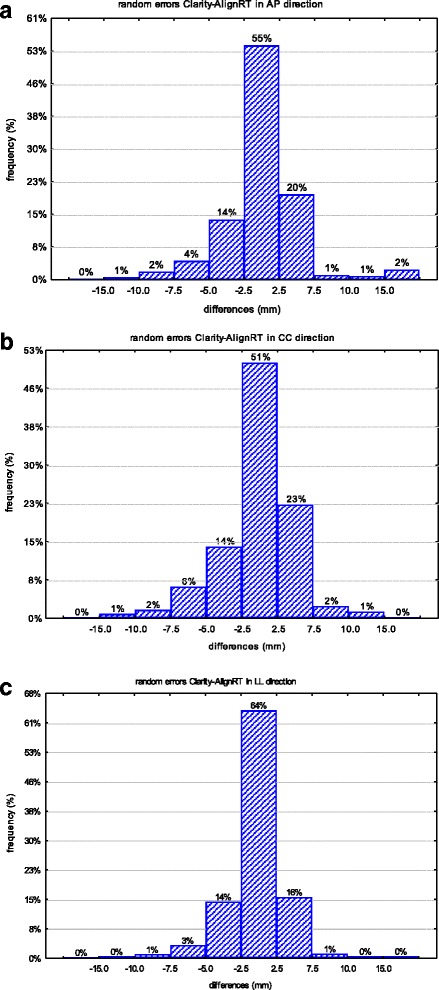
Histograms of the paired random errors differences between AlignRT and Clarity in AP (**a**), CC (**b**), and LL (**c**) directions

The box plot of the random errors differences between the two imaging modalities are reported in Fig. 3. The box plot shows that the 95% confidence interval (CI 95%) of the random inter-modalities discrepancies is inside the range from −7 mm to 7 mm, i.e. inside the expansion margin of PTV applied in AP direction in our series.

## Discussion

Intensity-modulation is becoming the standard for radiotherapy of the intact prostate. With this technique, tight margins should be applied to the target volume to limit the dose to normal tissues. It is therefore necessary, after the identification and correction of the systematic error, to accurately localize the target at each radiation fraction in order to minimize the risk of target missing related to the random error. For this reason, the use of imaging modalities without ionizing radiations can encourage the use of IGRT on a daily basis [14].

Over the last few years, a number of studies using video-surface imaging systems for patient setup verification have been published [7, 8, 14–19]. Some of them was performed on breast or intra-thoracic tumors and showed that surface imaging is a reliable method for patient position verification and may improve precision setup. Previous studies from our and other centres showed that AlignRT can be used also for the setup of prostate cancer patients [8–10], although the results showed some differences. Compared to portal images, Krengli et al. found mean systematic and random errors in the range of 1.2-2.0 mm and 0.3-0.7 mm, respectively [8]. Bartoncini et al. observed a concordance of portal and surface images in lateral direction for an error threshold of 3 mm and in the longitudinal and vertical directions for an error threshold of 5 mm [9]. Pallotta et al. observed mean positioning errors in the range of 0.1 ± 2.5 mm and 1.4 ± 4.0 mm between CBCT and surface images, with higher values in longitudinal and vertical axis and consequent potential improvement in 45% and worsening in 23% of patients by using surface imaging [10].

The potential of verifying the daily target position and analyzing inter-fraction setup errors by 3D-US imaging was investigated by other authors [20–25]. Most studies were performed by combining the use of different IGRT modalities. The combination of US, CBCT, portal images and electromagnetic transponders was analyzed by Mayyas et al. in 27 patients showing comparable (within 3–4 mm) inter-modality shifts [19]. A recent study analyzed 3 IGRT methods (US, stereoscopic X-ray imaging of implanted markers and kV CBCT) for prostate conformal radiation therapy in 186 patients [20]. The authors found that US guidance requires larger margins than the other IGRT methods, although they did not perform a true comparative analysis, since the 3 methods were applied to different patients’ cohorts. A larger PTV was suggested also by other authors who found US systems useful but less accurate than x-ray systems in terms of prostate localization [19–21]. In a recent study, Li et al. compared US and CBCT in prostate cancer patients with three gold markers. This study showed that 3D-US is comparable to CBCT. Using seed-match as reference, mean systematic errors of 3D-US were 1.3 mm, 0.8 mm and 1.4 mm, and random errors were 2.5 mm, 2.7 mm and 2.3 mm along lateral, longitudinal and vertical axes, respectively [11].

The present study, aiming at comparing daily variations obtained by two IGRT modalities is the first report on the analysis of the combined use of 3D-surface imaging and 3D-US in prostate cancer patients.

Our data on setup errors detected by AlignRT and Clarity are quite consistent with those reported by other authors [9, 22]. The asymmetric shape of the histograms and the non-zero mean value of the daily differences suggest a systematic component and possibly different sources of errors detected by the two imaging modalities (Fig. 1). The differences appear to be not only statistically significant but also clinically relevant since deviations larger than 10 mm were detected in relevant subset of the study population. The differences of systematic errors detected by the two different IGRT modalities were quite similar in AP and CC directions but significantly different in LL direction. As a matter of fact, an additional source of uncertainty in LL direction could be the precise identification of the lateral edge of the prostate by US.

The differences between the random errors detected by the two modalities were not statistically significant meaning that AlignRT measurements can be predictive of Clarity displacements after the adjustment for systematic errors. Looking at the distribution of the daily random error differences between AlignRT and Clarity, we found that they were generally small, picked around 0 and not exceeding the value of 7 mm in the 95% of the cases in all the directions, i.e. compatible with our standard requirements for PTV margins. This finding suggest that, after the correction for systematic errors, which can be verified by portals, Align-RT can be reliably used to monitor inter-fraction reproducibility with errors compatible with the PTV margins used in our clinical routine. In this case, we used 3D-US as reference cross modality but this observation can be extended to the cases where a CBCT imaging system is available. Usually CBCT is not performed every day during long treatments with standard fractionations and, in this clinical scenario, Align-RT could provide accurate surveillance of interfraction and also intrafraction positioning errors in a schedule of quality assurance by CBCT.

In our study, the concordance between the two IGRT modalities after correction for systematic error was found in condition of constant bladder filling as checked by 3D-US imaging at each treatment session. Of note, the differences of random errors detected by the two modalities in AP direction, which are most likely related to rectum filling, were quite relevant and almost statistically significant. These variations were not systematically checked in our study because undetectable by 3D-US imaging, but patients were recommended to have a daily enema to obtain rectum filling as reproducible as possible. Errors in AP direction detected by Clarity could be influenced also by a displacement caused by the probe pressure on the skin, as reported by other authors [23].

Based on our experience, 3D-surface and 3D-US imaging if complementary used, could represent a system to detect setup errors and organ motions without exposure to ionizing radiations and with a potential reduction of the cost of IGRT compared to the use of daily cone beam CT images [25–27].

Crucial aspects of the use of 3D-US imaging are the education and training of the professionals performing this technique. In our study, images were acquired by two radiation oncologists with experience in prostate management and specific training in US technique.

Our study has some limitations. Trans-abdominal US is a potentially efficient tool for daily targeting in radiotherapy; however, it has been associated with significant inter-user variability, with reports of acceptable images and acceptable alignments ranging from 68 to 97% [27–29]. In addition, when compared with prostate positioning on CT scans or implanted markers, the accuracy of trans-abdominal US can be questionable as reported by other authors [5, 19, 30]. Finally, the patient series was not systematically daily analyzed with CT images by CT-simulator or CBCT and the results cannot be validated with such a standard imaging modality, although data exist in the literature showing a consistency between 3D-US and CBCT data [22, 23].

## Conclusions

Daily variations detected by 3D-surface and 3D-US imaging in our series are in the range of the literature data. The error distributions for both imaging modalities were asymmetric, suggesting a systematic component with significant differences between the two imaging modalities. The systematic errors detected by 3D-surface and 3D-US imaging were significantly different only in the LL direction, possibly related to the difficulty in precise definition the lateral edge of the prostate by US. The differences between the random errors detected by the two IGRT modalities were not statistically significant, meaning that AlignRT measurements can be predictive of Clarity displacements after adjustment for systematic errors, given a constant bladder filling as verified in our study by 3D-US imaging. These findings suggest that the two techniques could be used as complementary QA methods in addition to weekly x-rays/cone beam imaging and could represent a daily “low-cost” and non-invasive IGRT modality for prostate cancer patients.

## Abbreviations

3D: Three dimensional
3D-US: Trans-abdominal ultrasound
AP: Anterior-posterior
CBCT: Cone beam computed tomography
CC: Cranial-caudal
CT: Computed tomography
CTV: Clinical target volume
EPID: Electronic portal imaging device
IGRT: Image guided radiotherapy
LL: Later-lateral
PTV: Planning target volume
SD: Standard deviation
TPS: Treatment planning system
US: Trans-abdominal ultrasound
